# The correlation of age and body mass index with the level of both protease MMP3 and anti-protease TIMP-1 among Indonesian patients with chronic obstructive pulmonary disease: a preliminary findings

**DOI:** 10.1186/s13104-018-3669-y

**Published:** 2018-08-02

**Authors:** Mulyadi, Sunnati, Mulkan Azhary, Faisal Yunus, Fariz Nurwidya

**Affiliations:** 1 Department of Pulmonology and Respiratory Medicine, Faculty of Medicine, Universitas Syiah Kuala, Banda Aceh, Indonesia; 2 Department of Periodontology, Faculty of Dentistry, Universitas Syiah Kuala, Banda Aceh, Indonesia; 3 Department of Pulmonology and Respiratory Medicine, Faculty of Medicine, Universitas Indonesia, Persahabatan Hospital, Jalan Persahabatan Raya No.1, Rawamangun, Jakarta, 13230 Indonesia

**Keywords:** Age, BMI, Salivary MMP-3, Salivary TIMP-1

## Abstract

**Objectives:**

Individuals with chronic obstructive pulmonary disease (COPD) are usually > 50 years of age and have a low body mass index (BMI). An imbalance between matrix metalloproteinases (MMPs), including MMP-3, and tissue inhibitor of metalloproteinase 1 (TIMP-1), play a role in tissue degradation of lung extracellular matrix among COPD individuals. The purpose of the present study was to correlate age and/or BMI with salivary levels of MMP-3 and TIMP-1 among Indonesian subjects with COPD.

**Results:**

Thirty COPD patients were recruited to undergo thorough physical assessment and saliva collection for evaluating TIMP-1 and MMP-3 levels using commercially available kits enzyme-linked immunosorbent assay method. The mean (standard deviation) participant age and BMI were 60.5 (8.13) years, and 23.1 (4.75) kg/m^2^, respectively. Furthermore, the mean (standard deviation) of TIMP-1 and MMP3 levels were 23.99 (6.85) ng/mL and 1.81 (1.167) μM, respectively. Age was negatively correlated with MMP-3 (*P* < 0.05), but not with TIMP-1 levels. Age and BMI were not correlated with TIMP-1 level (*P *> 0.05). Collectively, this study demonstrated that age has a negative correlation with the protease marker (i.e. MMP-3), but not the anti-protease marker (TIMP-1). BMI was not correlated with either protease/anti-protease marker among Indonesian subjects with COPD.

## Introduction

The proportion of active smokers with chronic obstructive pulmonary disease (COPD) is estimated to be 10–15% [1, 2]. COPD is characterized by partially irreversible airflow limitation, emphysema, reduced diameter of the small airways, and chronic bronchitis [3]. Smoking cessation may improve symptoms; however, the inflammatory process within the airways and in the lung parenchyma will persist [4].

Matrix metalloproteinases (MMPs) are counteracted by tissue inhibitor of matrix metalloproteinases (TIMPs), which results in reduced extracellular matrix (ECM) breakdown. The balance between MMPs and TIMPs is essential in maintaining the integrity of normal body tissues. An imbalance in the ratio of MMPs and TIMPs may manifest as numerous pathological conditions including rheumatoid arthritis, cancer, and periodontitis [5].

In the field of periodontology, smoking has been associated with an increased incidence and severity of various periodontal diseases [6]. Inflammation within the oral cavity and in the airway can be assessed using biomarkers. In the case of periodontitis, various tests can be performed using samples obtained from saliva and gingival crevicular fluid [7, 8].

TIMP-1 inhibits active MMPs, including MMP-1, MMP-3, and MMP-9. It has been reported that changes in MMP-1 poorly correlated with disease intensity and progression in COPD [9]. TIMP-1 binds to pro-MMP-9 to prevent the activation of pro-MMP-9. However, neutrophil elastase acts by dissociating the binding of TIMP-1 to pro-MMP-9, which allows MMP-3 to activate pro-MMP-9 to become MMP-9 [10]. Our previous study revealed that salivary MMP-9 was not correlated with lung function in COPD patients, which prompted us to confirm whether salivary MMP-3 could be a viable biomarker associated with age and body mass index (BMI) at the onset of COPD [11]. This study aimed to investigate possible correlations between degenerative variables, such as age and BMI, and the level of salivary MMP-3 and TIMP-1 in Indonesian patients with COPD.

## Main text

### Methods

This study recruited 30 consecutive smokers with COPD who visited the pulmonary outpatient clinic of the Dr. Zainoel Abidin General Hospital, located in Banda Aceh, Indonesia. The inclusion criteria were a diagnosis of COPD; current smoker; male sex; > 50 years of age; and 20 pack-years of cigarette consumption. Diagnosis of COPD was established according to spirometry results (i.e., forced expiratory volume in 1 s/forced vital capacity ratio < 70% according to the 2017 Global Initiative for Chronic Obstructive Lung Disease guidelines [12]. Patients with respiratory or oral infections, or malignancy, were excluded. This study was approved by the Ethics Committee of the Faculty of Medicine, University of Syiah Kuala, Banda Aceh, Indonesia (Ethical Clearance No. 197/KE/FK/2013). All participants were required to provide informed consent before undergoing the research procedures.

Saliva was collected into sterile pots and stored in a − 80 °C freezer for analysis of TIMP-1 and MMP-3 using a human TIMP-1 quantikine ELISA kit (R&D Systems, Minneapolis, MN, USA) and Sensolyte 520 Generic MMP fluorimetric assay kit (AnaSpec, Fremont, CA, USA), respectively, according to manufacturer’s protocols.

Data are presented as mean and standard deviation (SD). Pearson’s correlation coefficient was used to determine the correlation between variables. The data were analyzed using SPSS version 15.0 (IBM Corporation, Chicago, IL, USA) for Windows (Microsoft Corporation, Redmond, WA, USA), and *P* < 0.05 was considered to be statistically significant.

### Results

The mean (SD) age of the subjects was 60.5 (8.13) years, and the mean (SD) BMI was 23.1 (4.75) kg/m^2^. The mean (SD) level of salivary TIMP-1 was 23.99 (6.85) ng/mL, and the mean (SD) activity of salivary MMP-3 was 1.81 (1.167) μM (Table 1).

**Table 1:** Subject characteristics

Variable	Mean (SD)
Age (years)	60.5 (8.13)
Body mass index (kg/m^2^)	23.1 (4.75)
MMP-3 activity (µM)	1.81 (1.167)
TIMP-1 level (ng/mL)	23.99 (6.85)

*MMP-3* matrix metalloproteinase-3, *TIMP-1* tissue inhibitor of metalloproteinase 1

Next, the authors analyzed the correlation between age and BMI with MMP-3 activity using Pearson’s correlation coefficient. Age was significantly correlated with the salivary MMP-3 levels (*P* = 0.002), however BMI was not correlated with MMP-3 activity (Table 2).

**Table 2:** Correlation between individual characteristics and MMP-3 activity

Variable	MMP-3 activity	
	R	*P* value
Age	− 0.42	0.02
Body mass index	− 0.01	0.97

*MMP-3* matrix metalloproteinase-3

Finally, the authors investigated possible relationships between BMI and anti-protease (i.e., TIMP-1) level using Pearson’s correlation coefficient, and found that BMI was not correlated with salivary TIMP-1 levels (Table 3).

**Table 3:** Correlation between age and body mass index and TIMP-1 level

Variable	TIMP-1	
	R	*P* value
Age	< 0.001	0.99
Body mass index	0.25	0.19

*TIMP-1* tissue inhibitor of metalloproteinase-1

### Discussion

Identification of protease and anti-protease biomarkers in COPD patients using non-invasively obtained fluid samples are under intensive investigation. It has been reported that TIMP-1 levels in lung tissue increase in response to higher activities of lung parenchymal MMP-3 [13]. In periodontitis, the development of attachment loss will be inhibited by TIMP-1 to prevent further potential tissue damage. These are considered to be the natural responses of anti-proteases against increased protease activity [14].

MMPs are divided into several subgroups according to their primary substrate, such as collagenases (MMP-1 and MMP-13) degrading the interstitial collagens, and gelatinases (MMP-2 and MMP-9) that degrade basal membrane components such as fibronectin and elastin [15]. As an “anti-protease”, TIMP-1 acts in response to increased activities of MMP-3 and MMP-9, and acts to prevent degradation of ECM in tissues. If there is reduced ability of TIMP to act against exogenous and endogenous MMP, this will result in the prolonged and increased activity of MMP-3 and MMP-9, which results in ECM damage [16, 17].

MMP-3 is a broad-spectrum MMP that functions by activating latent MMPs including pro-MMP-1, -8 and -9. The overall cascade of tissue degradation occurs due to the involvement of the regulatory effects of MMP-3 both physiologically and pathologically. The expression of MMP-3 and increased amounts of MMP-3 messenger RNA in periodontal lesions have been reported in previous studies, which concluded that MMP-3 may be marker for assessing the tissue degradation process [5].

There is an increased risk for periodontitis among smokers with COPD. In contrast, smokers without COPD exhibit higher mean TIMP-1 levels in response to increased activities of MMP-3 and MMP-9. Verstappen et al. [18] observed increased levels of TIMP-1 in the healthy periodontium compared with inflamed periodontium, which represents a homeostatic mechanism to sustain balances between proteases and anti-proteases to prevent ECM damage.

TIMP-1 is crucial to the balance of increased MMP-9 activity, and cigarette smoke exposure among smokers without COPD could be implicated in the activation of several pro-inflammatory signally pathways, including oxidative stress, pro-inflammatory cells, and alveolar macrophages, which results in the activation of several proteases, especially MMP-9. An in vivo study reported that TIMP-1 was highly expressed in respiratory epithelial cells that had been exposed to chronic inflammation. The epithelial tissue expressed TIMP-1 in response to the activity of MMPs [19]. Furthermore, in a study by Higashimoto et al. [20], involving smokers without COPD who consumed the same number of cigarettes as smokers with COPD, suggested a similar risk for continuous airway inflammation between both groups and due to persistent cigarette smoke exposure.

Fujita [21] found that the activities of both MMP-9 and TIMP-1 following lung injury in COPD led to either recovery or degradation of ECM. Among smokers without COPD, the activities of MMP-9 and TIMP-1 will result in recovery and resolution; meanwhile, in smokers with COPD, this will result in ECM destruction and emphysema [21].

In conclusion, our preliminary findings suggest that salivary TIMP-1 is not a suitable biomarker in Indonesian subjects with COPD. There was, however, a significant negative correlation between age and the level of MMP-3 activity. Further study with a more heterogeneous population is required to clarify the precise role of TIMP-1 as a possible biomarker of anti-protease activity in the lung.

## Limitations

The current study involved a small number of subjects; therefore, the findings may not reflect the true correlation that may exist between protease/anti-protease imbalances and age and BMI. Furthermore, the study was conducted in a single-institution and involved only male subjects, which, considering the heterogeneity of the Indonesian population, may restrict the generalizability of the findings.

## Abbreviations

BMI: body mass index
COPD: chronic obstructive pulmonary disease
ECM: extracellular matrix
MMP: matrix metalloproteinase
TIMP: tissue inhibitor of metalloproteinase
